# Miniaturized Transcatheter Leadless Pacemaker in a Patient with Double Mechanical Prosthesis

**DOI:** 10.5935/abc.20160192

**Published:** 2017-03

**Authors:** Marta Pachón, Alberto Puchol, Finn Akerström, Andrés Sánchez-Pérez, Miguel A. Arias

**Affiliations:** Cardiac Arrhythmia and Electrophysiology Unit - Department of Cardiology - Hospital Virgen de la Salud, Toledo - Espanha

**Keywords:** Pacemaker, Artificial/adverse effects, Heart Valve Prosthesis, Arrhythmias, Cardiac/therapy

## Introduction

Despite technical advances and accumulated experience, complications continue being a concern for patients implanted with permanent pacemakers. Several leadless pacemaker systems have now been developed in order to reduce the rate of complications in patients implanted with conventional transvenous pacemaker.

## Case Report

**Figure 1:**
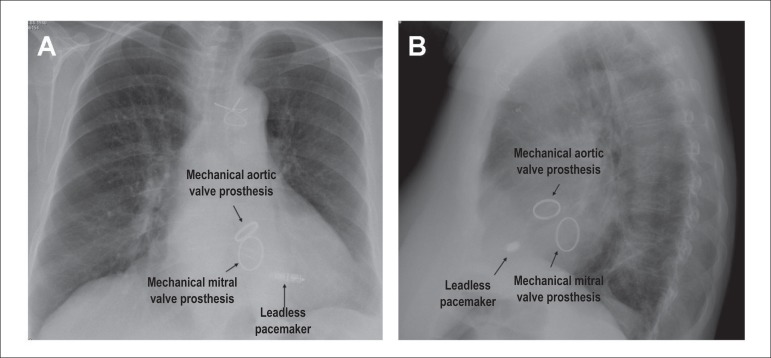
Chest X-Ray of the patient (panel A, posteroanterior view; panel B: lateral view) after leadless pacemaker implantation.

A 75-year-old female patient with a background of systemic arterial hypertension, chronic atrial fibrillation with an episode of peripheral arterial embolism in the right upper limb and rheumatic valve disease, underwent mitral and aortic valve replacement receiving two mechanical valve prosthesis in combination with left atrial appendage occlusion. Six months after surgery, pharmacologic therapy to achieve heart rate control was very difficult and inadequate and the patient was scheduled for permanent pacemaker implantation. In order to avoid lead or pocket complications, the Micra transcatheter leadless pacemaker (Medtronic Inc., Minneapolis, MN, USA) was implanted through the femoral vein using a steerable catheter delivery system with the use of a 23-French introducer. The procedure was performed under uninterrupted acenocoumarol therapy with therapeutic international normalized ratio (INR 2.5). Sedation and local anesthesia was applied and the implant was successful upon initial device positioning at the mid-septum of the right ventricle with no complications. Access site closure was performed using a subcutaneous venous figure-of-8 suture. The pacing capture threshold at implant was 0.38 V measured at 0.24 ms, the R-wave sensing amplitude was 8.8, and the pacing impedance was 730 ohms. There were no complications and the patient was discharged home the next day after chest X-ray showed the device was positioned perfectly (Figure 1) and electrical pacing parameters were appropriate. At three months of follow-up the patient has shown no complications and the pacing capture threshold was 0.38 V at 0.24 ms, the R-wave sensing amplitude was 9.2 and the pacing impedance was 680 ohms.

## Discussion

In spite of technological advances and the enormous accumulated experience, conventional pacemaker therapy continues to be associated with a great variety of potential complications either in the short and long-term.^1^ They are particularly related to the device (hematoma, skin erosion, pocket infection) or as a result from transvenous lead placement (pneumothorax, cardiac perforation, lead dislodgement, venous occlusion, loose connector pin, conductor lead fracture, insulation lead break, infections, tricuspid valve damage, etc.). Early performance and safety data for the Micra transcatheter leadless pacemaker are positive^2,3^ and leadless pacemakers represent a promising alternative for many patients, eliminating the main sources of complications associated with conventional transvenous pacemaker implantation.

## Conclusion

Patients with mechanical heart valve prosthesis might represent a subgroup of patients for whom this new therapy can bring higher benefits due to the need for lifelong anticoagulation and the serious consequences of permanent transvenous pacemaker system infections.
